# Holiday Club Programmes in Northern Ireland: The Voices of Children and Young People

**DOI:** 10.3390/ijerph18031337

**Published:** 2021-02-02

**Authors:** Jackie Shinwell, Ellen Finlay, Caitlin Allen, Margaret Anne Defeyter

**Affiliations:** 1 Healthy Living Lab, Coach Lane Campus, Northumbria University, Benton NE7 7XA, UK; j.shinwell@northumbria.ac.uk; 2 Children in Northern Ireland, Belfast BT6 9HL, UK; ellen@ci-ni.org.uk (E.F.); callen@effectiveservices.org (C.A.)

**Keywords:** holiday hunger, food insecurity, holiday provision, Northern Ireland

## Abstract

In Northern Ireland, nearly 30% of children are thought to be at risk of going hungry in the summer holidays when they are unable to access free school meals. Community groups, voluntary groups, local authorities, and faith groups have responded to this concern by developing and delivering holiday programmes that enable children from low-income families to take part in activities and access food. The current study used purposive sampling to investigate children’s and young people’s views of holiday provision, from across three holiday clubs, in Northern Ireland. Both primary school children (*n* = 34; aged 4–11) and secondary school children (*n* = 31; aged 12–17) showed high levels of awareness of poverty and food insecurity and associated pressures and stresses on households. Importantly, children and young people did not feel stigmatised about attending holiday provision, suggesting a positive and inclusive culture towards holiday club attendance. Children reported that they enjoyed the range of activities provided at holiday clubs and reported that attendance improved their self-confidence, especially for some older children, who acted as peer mentors to younger attendees, helped them to develop new skills, and provided them with opportunities to socialise with peers in a safe environment, out with their normal social groupings in school. Older children showed a high level of shrewdness and knowledge of sectarian divides in communities but spoke positively about how different religious or cultural backgrounds did not matter in terms of meeting and making new friends in holiday club settings. In terms of food provision, the findings of this study suggest that further work needs to be done to support children to access and eat healthy, nutritious food.

## 1. Introduction

In Northern Ireland, nearly 30% of all school-aged children are entitled to a free school meal during term time [1]. However, during the school summer holidays, when the safety net of free school meals is removed, many children from low-income families are thought to be at risk of holiday hunger [2]. Holiday hunger has been defined as “the tendency for children to be unable to access an adequate supply of nutritious food during the school holidays” [3] (p. 2). In response to concerns that nearly 96,000 school-aged children in Northern Ireland may be at risk of going hungry in the holidays, more than 80 organisations, including voluntary and community groups, local authorities, schools and churches, have developed summer holiday clubs to enable children from low-income families and newly arrived immigrant families to access food and activities during the eight week school summer holiday period [4].

Prior research suggests that access to food is an important aspect of holiday club attendance and holiday club staff have reported that holiday clubs are needed because of high levels of food insecurity in disadvantaged communities [3,5]. Moreover, parents and carers have reported that holiday club attendance may reduce household food insecurity because food at home lasts longer when children are provided with a mid-day meal at holiday club [6]. Recent research has shown that the benefits of holiday club attendance extend beyond the provision of food. Clubs reduce social isolation in children and children are able to take part in a variety of physical, creative and potentially educational activities that they otherwise would not have been able to do if they had not attended holiday club [3,7,8]. The majority of qualitative studies regarding the benefits of holiday provision have been conducted with adults, and the views of children who attend holiday clubs are under-represented in the literature. To our knowledge, only one peer-reviewed study has investigated the views of children who attended breakfast holiday clubs in the UK [8]. The authors found that children aged 4–15 years of age valued their holiday breakfast clubs as they provided opportunities to meet friends, participate in a range of activities, and access to a free breakfast meal, although there was no suggestion from children that they were attending holiday club due to household food insecurity. There is growing evidence that young people want a greater say in shaping decisions that affect their lives [9,10]. Although recent studies have furthered our conceptual understanding of potential differential effects of community engagement in public health interventions [11], few studies have included the voices of young children. The aim of the current study, therefore, was to extend the research by Defeyter, Graham and Prince (2015) [8] by exploring the implementation, uses and potential benefits of holiday clubs through the voices of children and young people in the unique setting of Northern Ireland that has its own rich cultural and social history that is distinct from the rest of the UK.

## 2. Materials and Methods

### 2.1. Approach

This study employed a qualitative design. Semi-structured interviews were used to collect data in focus groups with child holiday club attendees in this study. This approach was adopted as focus groups are inclusive and allow participation regardless of age and educational ability and provide an environment where children may feel able to speak freely and openly in the company of their peers with whom they will be more familiar [12]. Focus groups were held on holiday club premises during the hours of operation of the holiday club as this was considered the most appropriate way of collecting data.

### 2.2. Participants

Purposive sampling was used to recruit participants to this study. Purposive sampling refers to the method of recruiting participants to a study who are well placed and knowledgeable about the subject matter being researched [13]. Participants were recruited from three of the four holiday clubs that operated during summer 2019 that were part funded by Children in Northern Ireland (CiNI), a charitable body that seeks to work with a range of organisations to ensure that children’s interests are reflected in public policy in Northern Ireland. Club 1 was delivered by a charity and took place in a school that was hired for the duration of the summer holiday club. Some attendees lived locally, but the majority travelled to the club by bus, which was provided free of charge to holiday club attendees. There was no charge for attending the holiday club. Club 2 operated out of two community-based sites. The main site was a purpose-built youth centre and was owned by the club. The second site was a local authority owned community centre a short distance away. Attendees lived within a three-mile radius of the sites and either walked to the club or were dropped off by parents. There was a cost of £2 per day for attending the holiday club, but this was waived for children and young people who had been referred to the club by social services or the child’s school. Club 3 was held in a purpose-built community centre and children lived nearby and either walked to the club or were dropped off by parents. The cost of attending this club ranged from £1 to £5 per day, depending on the activities on the day.

Across the three clubs, all attendees (*n* = 65) were approached to take part in this study, and all agreed to participate in one of eighteen focus groups. The number of participants in each focus group varied between two and six children and depended on the number of children who were available on testing days. Demographic data on age and gender were self-reported by participants and are presented in Table 1. Characteristics of each of the holiday clubs (*n* = 3) are presented in Table 2.

### 2.3. Materials

Letters of invitation, research information sheets and consent forms pertaining to the research were developed for child holiday club attendees, their parents and carers and holiday club staff. A semi-structured schedule was prepared to guide interviews (see Table 3) which comprised a series of questions which could be drawn upon by the researcher if needed, to encourage children to talk freely about their experiences relating to the CiNI summer 2019 holiday programme.

### 2.4. Procedure

Following ethical approval from the Faculty of Health and Life Sciences Ethics Committee at Northumbria University (reference no: 17110), the CiNI administration team was asked to disseminate information about the research study to the holiday club leaders of the four holiday clubs in the 2019 summer programme. Three of the four club leaders expressed an interest in taking part and were then sent a research information pack including a letter of invitation, an information leaflet, and an opt-in consent form. Once consent was received, club leaders disseminated research information packs including a letter, research information sheet and a parental opt out consent form to parents and carers. An interpreter verbally explained the parental consent forms to parents who spoke little or no English. All parents agreed to their child’s participation in this study. On each day of data collection, children were approached by a researcher and were invited to take part in this study. Each child was provided with a copy of a child-friendly research information sheet, and this was read aloud to each child and young person, individually, to seek verbal consent prior to data collection. Children and young people were reminded that they did not have to participate in this study and were free to withdraw from the focus group, and this research study, at any time. At this stage, no children or young people opted out of this study. Prior to the commencement of focus groups children were asked to self-report their age and gender. A few children did not speak or spoke very little English. For their views and experiences to be incorporated into this study, an interpreter accompanied these children to the focus groups and interpreted children’s verbal contribution in real time. Focus groups were, on average, 20 min in duration. At the conclusion of each focus group, a child-friendly debrief sheet was read aloud to the group, and children and young people were also given a copy to take home. All focus groups were audio recorded and orthographically transcribed verbatim for subsequent thematic analysis.

### 2.5. Data Analysis

Transcripts were read several times in order to gain a thorough overview of the data. All data were uploaded into NVivo 12 for ease of access and organisation. The data from focus groups were analysed according to two age groups: primary school age children (4–11 years of age) and secondary school age children and young people (12–17 years of age). Data from interviews were coded and analysed in accordance with guidelines on thematic analysis produced by Braun and Clarke (2006) [14]. Quotes were grouped under topic headings and main themes and associated subthemes were identified within the data.

## 3. Findings

Children reflected on and shared their views on why holiday club provision was needed, the benefits of holiday club provision and the type of activities and food provided in holiday club settings in Northern Ireland. Four broad themes were identified across primary school and secondary school-aged children and young people: (1) the need for holiday clubs; (2) the benefits of holiday club provision for children; (3) the benefits of holiday club provision for parents; and (4) operational characteristics of holiday clubs. A fifth theme regarding ‘The benefits of holiday club provision for the individuals, families and the community’ was identified from the focus groups with secondary school-aged children and young people only. A summary of the main themes and associated sub-themes are presented in Table 4. This is followed by a detailed analysis of each theme together with sample quotes to illustrate the findings from both primary and secondary school-aged children and young people. In order to clearly indicate the source of each quote, each quote is followed by a number (the number of the focus group, range 1–18) and a letter (P = primary school-aged participant, and S = secondary school-aged and above participant).

### 3.1. Main Theme 1: The Need for Holiday Clubs

Both primary school-aged children and secondary school-aged children were able to identify the need for holiday provision and two sub-themes were identified: holiday hunger and family circumstances.

Some children told the researchers that they were aware of “holiday hunger” and that holiday clubs were needed because some children, particularly those who normally received free school meals during term time may be at risk of going hungry in the holidays. However, attendance at holiday club meant children were able to access food during the school summer holiday. Older children suggested that parents may try to protect their children from the financial difficulties that their families may be experiencing, as reflected in the following discussion in one focus group of older children:

Child 1: “well, the whole point of [name of club] is to give kids that get free school dinners, well mostly it’s to make sure that they eat during the holiday period, but also to give them something they can do and learn how they can take better care of themselves and stay healthy and all that and also like, it just gives them something to do over the holidays”

(CFG 3S)

Child 2: “yeah I think you can really tell just by sitting with the children because holiday hunger can happen at home so like when they come here food is provided, there’s like options for them and like make sure that every child eats something so it’s not like you can tell anything”

(CFG 3S)

Child 3: “no I also reckon a lot of them don’t really realise because you have the ones that obviously may have issues at home, but they will still be picky about what they eat. I think they just don’t really realise their parents are the ones scraping by for them to eat but they don’t, they are just kids so they’re just living”

(CFG 3S)

However, some children and young people indicated that they were aware that they and others were referred to the holiday club because of their family circumstances, as explained by a secondary school-aged child:

“…me and my sister don’t pay the £2 because my mum and my dad are split up and things like that, but I think if my mum had to pay that on top of everything else it would be a bit stressful and she [the sister] wouldn’t be coming all the days that she does, but even I don’t think mum would mind paying it because it gets my sister out”

(CFG 5S)

Children and young people also showed a great deal of empathy for children who may be experiencing hunger and thought that provision of food at the holiday club without an additional charge over and above any cost of attendance meant that everyone was the same and nobody was left out, as demonstrated in the following exchange between primary school-aged children:

Child 1: “yeah everyone gets it.”

(CFG 10P)

Child 2: “no one is left out; they make sure everyone gets something”

(CFG 10P)

Child 3: “even if somebody doesn’t like the food that they provide, they still make sure that they would cook something that they like.”

(CFG 10P)

Likewise, secondary school-aged children also considered that the provision of food without an additional cost made everyone equal:

“and just like it puts everyone on an equal plain because some kids might have better food than other kids that they could get picked on for something like that”

(CFG 3S)

Children felt that the change to providing food to all, with no additional cost was a positive change to the prior food delivery model. A primary school-aged child recalled the previous year’s summer holiday club when attendees had to buy food from the club tuck shop or bring a pack lunch. She had been aware of children who may have been embarrassed because they had no money to buy food from the tuck shop and how she would share her money with them:

“I’d usually just help them, like give them money to help them out instead of letting them sit and struggle if they’re really hungry, then I just give them money too”

(CFG 10P)

### 3.2. Main Theme 2: Benefits of Holiday Provision for Children and Young People

Five sub-themes were identified regarding the benefits of holiday club provision for children and young people: the range of activities available in holiday clubs; the acquisition of new skills and building self-confidence; having fun, making and sustaining friendships; a safe place to play and the alternatives to non-attendance. These sub-themes were common across both age groups.

Children and young people told researchers that they enjoyed attending holiday club because they could participate in a range of activities that they would not be able to do or have permission to do at home. In one club for example, children said they liked taking part in the kitchen activity because they were able to get more involved in food preparation compared to home, as one primary school-aged child explained:

“well, we get to put in all the ingredients and stuff like the adults and do all the, because my aunty usually puts in the most ingredients, because I love baking with my aunty, and I do the least ingredients”

(CFG 1P)

As a result of the range of activities on offer, many children reported that they gained new skills, for example, CPR that might come in useful:

“yeah, so then we know better in the future if anything happens then we know what to do”

(CFG 1P).

Some skills complemented learning that had taken place in school and at the community clubs children attended during the school year. A number of older children, specifically those who volunteered as peer mentors, reported increases in their self-confidence. These children also gained a recognised qualification, in this case the Open College Network qualification in Youth Leadership. This was summed up by one volunteer peer mentor who said:

“it’s definitely helped with, personally it has helped with my confidence because when I was first here, I was like “I can’t lead a session I can’t be talking to people, like children, they’re not going to listen” but I’ve definitely noticed a change in myself where I can get the kids attention, get things done.”

(CFG 3S)

However, improvements in self-confidence, through participation in a variety of activities, were also evident in primary school-aged children. A primary school-aged child reported that participating in drama classes had boosted her self-confidence, and this in turn had helped her make new friends:

“yeah, because I used to be really scared to make new friends and this club really helps you make new friends and now, I’m more confident to make new friends”

(CFG 10P)

The affordance of holiday clubs to enable children to make new friends, in a safe environment, was an important aspect of attending holiday club for many children. For instance, one child said:

“we come here, and we meet new people, like this is the first time we met [name].”

(CFG 4S)

Another child said:

“for me I was like, I wasn’t confident enough to leave the house sometimes, I was always, I would always get bullied and be called ‘speckky’ and all so I would and here no one is like that, they’re all really nice people”

(CFG 10S)

When children were asked to consider what they normally do when not at holiday club, some primary school-aged children reported that they would spend time playing in the park with family and friends. Other children said that relatives would care for them: *“I’m going to granny’s; I’ll go back to granny’s” (CFG 12).* However, numerous children from across both age groups told researchers that without holiday club, they would be sitting at home bored with nothing to do. Younger children suggested they would spend their time lying about or watching TV:

Child 1: “I’d be watching TV.”

(CFG 16P)

Child 2: “bored in the house, just lying about.”

(CFG 16P)

Some older children indicated that without the holiday club, their summer would largely be spent indoors playing on computer games:

Child 1: “I’ve came along to this programme because I’ve been at this youth club years and it’s an experience working with the young kids in the morning as a volunteer and if I wasn’t here, I would be sitting in the house playing on the play station.”

(CFG 8S)

Child 2: “if I wasn’t here, I would be doing the exact same, that’s the bad bit but it (holiday club) gives you something to do and it’s an excuse to go outside.”

(CFG 8S)

One primary school child said they did not have any games at home, and another child reported that, when given the option of staying at home with their parent, they preferred to attend holiday club:

“like there is fun stuff to do so I just like going here and sometimes my mum says you can just stay with me and I’m like nah I’ll go (to holiday club)”

(CFG1P)

Some older children who attended evening sessions at their holiday club said that if it were not for holiday club, children and young people would be hanging around on the street: ***“****it does keep kids off the street” (CFG 9S)* and told researchers that if they were not at holiday club they would on the hanging around the streets and/or drinking:

Researcher: “yeah so what else would you be doing if you weren’t here?”

Child 1: “sitting in the house”

(CFG 9S)

Child 2: “hanging round the streets”

(CFG 9S)

Child 3: “drinking.”

(CFG 9S)

### 3.3. Main Theme 3: Benefits of Holiday Provision for Parents

Four sub-themes were identified in focus groups with children and young people regarding the benefits of holiday club provision for parents/carers, namely the expense of the summer holidays; how holiday club saved parents/carers money; reduction in parental anxiety about food and providing respite for parents/carers.

Children and young people in both age groups were aware that the summer holidays were an expensive time of year for parents and one primary school-aged child explained that *“the best thing about it [holiday club] is it’s all free” (CFG 10P)*. Moreover, for parents who worked, holiday club offered a much cheaper alternative to other childcare provision, as suggested by a secondary school-aged child:

“it’s only £2 so I think it’s a lot less than getting someone to baby sit while you’re at work and having to go for them long hours when they can come here for the two hours and do loads of fun stuff.”

(CFG 5S)

Children were also aware that attending holiday club alleviated their parents’ anxieties about feeding their children during the school summer holiday. A primary school-aged child explained that her parents would be less worried because food was provided at holiday club:

“yeah, because mainly my parents are worrying if I if I’ve got something to eat but now, they know that I can get something here”

(CFG 16P)

The same opinion was shared by secondary school-aged children who also added that provision of food at holiday club meant that parents and carers did not need to worry about providing a pack lunch:

Child 1: “yeah because they don’t have to worry about oh what are they going to have to eat when they’re at the summer scheme.”

(CFG 9S)

Child 2: “yeah, it’s like we don’t have to like oh do I need pack lunch, there’s always food there for them.”

(CFG 9S)

A primary school-aged child also explained that if she and her brother ate at holiday club, this would relieve the stress for their mother of having to cook when they got home:

“I like it because it doesn’t stress mummy us coming home from club and mummy having to go and make dinner, so with them providing us with food that means me and my brother don’t have to go home and say we’re hungry, she doesn’t have to cook dinner ‘til like 7 or 8 o’clock and the toasties as [name of child] was saying, the pizza is amazing, it’s just really good that they provide us with food”

(CFG 10S)

An older child who volunteered as a peer mentor expressed a similar sentiment and commented that the money parents saved on buying food could be used for other things:

“it gives their parents a bit of like relief, so they know that if they are going to come here they are going to get fed and they don’t have to worry about getting food for them and going out and having to spend money where they maybe need to spend somewhere on something else maybe, not more important than eating like you know what I mean.”

(CFG 8S)

In addition to helping parents cope with the expense of the summer holidays, holiday clubs benefited parents in other ways. For example, one primary school-aged child explained that a consequence of her and her brother [who had special needs] attending the holiday club meant her parents got some respite and *“basically they get some peace and quiet*” (CFG 14P). Other children said parents could tidy the house or do other things, as suggested by a secondary school-aged child:

“yeah, it gives their parents time to like if they need to do something about the house or maybe go for their shopping it gives them time to do it while their kids are here”

(CFG 8S)

Or, as one primary school-aged child suggested, parents could spend time alone when children were at holiday club:

“well, I think my mummy spends time by herself when I come here.”

(CFG 13P)

### 3.4. Main Theme 4: Operational Characteristics of Holiday Clubs

Three sub-themes were identified regarding the operational characteristics of holiday clubs: type of activities offered, length of provision and hours of operation and food.

It was clear that children had strong, positive feelings about their holiday club and how it made them feel, with children saying that compared to previous summers, attending holiday club made them feel *“happier” (CFG 1P)* and that *“it makes it (summer) a bit better” (CFG 1P).* When children were asked if there was anything that could be done to improve holiday club, children indicated that they were generally happy with their club. Where improvements were suggested, these included having more breaks, more free play, increasing the number of out of the holiday club venue activities, including trips and the opportunity for residential trips, and for holiday clubs to be open for more weeks during the summer. For example, primary school children who attended a club that was open three days a week for three weeks of the holidays said that they would have preferred it if the club was open for the whole week and for more weeks during the summer:

“yeah, like it could be for a month and then a week in August like say for three weeks”

(CFG 1P)

And:

“I’d like the whole week; the club is better than sitting at home”

(CFG 1P)

Similarly, older children indicated that they would like clubs to be available for each day of the summer school holiday, as reflected in the following exchange between secondary school-aged children:

Child 1: “Longer, it needs to be longer”

(CFG 6S)

Child 2: “It needs to be all summer”

(CFG 6S)

Child 3: “Later hours as well”

(CFG 6S)

Child 4: “Like more hours”

(CFG 6S)

When children and young people were asked what they thought of the food that was provided in holiday club, this elicited numerous responses. They acknowledged that the food provided accommodated children with special diets, such as halal or vegetarian. For instance, a primary school-aged child said:

“and it’s hard for like the Syrians just coming in and they can’t eat the same like ham as all of us and then if say, if say, and all the one that they eat is gone, but then as [name of person] always said, that they make sure that everyone gets some so they would make more for them”

(CFG 10P)

Some secondary school-aged children indicated that they liked the food and that their dietary requirements were met even though this might make things more complicated for kitchen staff, as demonstrated in the following exchange:

Child 1: “I get my own special food.”

(CFG 3S)

Child 2: “same.”

(CFG 3S)

Child 3: “I’m vegetarian so I’m making everything more awkward for the kitchen.”

(CFG 3S)

Older children who acted as mentors were aware that in previous years, some of the food provided in the holiday club had been donated from foodbanks, and perishable foods were difficult to store. This had, however, led to repetition in the type of food served: *“yeah previous [club name] have been like pasta, then like a different type of pasta” (CFG 3S).* They were aware that a lot of work had been done to improve the food offer: *“I think they have built up the variety now”* [CFG 3S] but added: *“the kids were still complaining” (CFG 3S).* This was reflected in interviews with younger children in the same club who said they did not like the food that was provided. A number of the children reported that on one particular day when homemade soup had been served for lunch, they had just eaten the bread: ***“****nope, I had loads of bread because I didn’t like it, but I had all the dessert” (CFG 1P)*. When asked what could be done to improve the food, children said that they would have liked more choice:

“maybe they could like make wee menus with like three or four options and then you could like pick and then at the bottom, it would, at the bottom of the menu, it could say if you really didn’t actually like any of those you could get like ham sandwiches or something like that”

(CFG 2P)

When asked what kind of food they would like to have for lunch, some children said that they wanted more meat or that the food they got at home was better, or they would have preferred pizza. In contrast, in clubs where snacks had been served, children were effusive in their praise of the ham and cheese toasties and sausage roll and chips:

“the toasties are just amazing, ham and cheese oh man, that’s all I can say”

(CFG 10P)

### 3.5. Main Theme 5: Benefits of Holiday Provision for Individuals, Families and Communities

Sub-themes identified in focus groups with secondary school-aged children regarding the benefits of holiday club provision for individuals, families and communities related to community cohesion, removing barriers between groups in society and supporting child immigrants.

Secondary school-aged children perceived that holiday clubs benefitted communities in multiple ways. For example, at holiday club, children made new friendships with children who lived in the same community but attended different schools, as explained by one focus group participant:

“they can get to know different people instead of the same people that they see in school every day and they make new friends and have fun basically”

(CFG 8S)

However, the impact of making new friends went beyond just a one-to-one benefit. Older children acknowledged that the simple act of mixing with people from different schools may benefit the community not only in terms of Northern Ireland’s historical segregation between Catholics and Protestants, but also in relation to newly arrived immigrant populations in the country. It was important, as explained by one young person, to remove barriers between different religious beliefs when children were young:

“it’s better to break them down when you’re younger than having to do it when you’re older, it’s a lot easier when you’re younger.”

(CFG 5S)

Removing barriers between children may ultimately benefit communities, as suggested by a secondary school-aged child:

“because even like going outside and playing, it gives the kids, there won’t be sort of like a tension, because sometimes there’s a religious tension sort of thing over here in [name of place] between like Protestants and Catholics, there’s sort of like that barrier, but I think with this, because it, you don’t really get to know who is Protestant and who’s Catholic and then there’s some other religions, and I think they just go over that barrier and just mix together”

(CFG 4S)

Children considered that removing barriers was particularly important because of Northern Ireland’s history, as one young person who acted as a peer mentor explained, segregation still existed in Northern Ireland: *“considering the segregation and it’s pretty segregated still, it’s pretty important,” (CFG 3S).* However, children and young people were of the opinion that an individual’s background or religion did not matter, and holiday club provided an environment where friendships could be formed regardless of background. This was reflected in the ethos of the holiday clubs, as one young person who volunteered as a peer mentor explained:

“I think one of the main things that is a bit different for [name of club] and their programmes and other youth clubs that I’ve been to, is that they don’t make it like the main focus, so this is like a Protestant person and this is a Catholic person, be friends, it’s just like so you are different, they just let people exist.”

(CFG 3S)

Children gave parents credit for allowing their children to attend a mixed holiday club:

“I think that’s also a big step for the parents because I know some people just wouldn’t let their children go here, it’s like there’s that religious gap but then the parents have taken a step forward.”

(CFG 4S)

Children also spoke of the benefits of mixing with children from different countries particularly because some children who attended holiday clubs were newly arrived in Northern Ireland. This had a number of benefits including promoting understanding of different cultures:

“it gives them more experience because we all come from different backgrounds, it’s better to know how to treat others that aren’t just the same as you”

(CFG 8S)

Children described how without holiday clubs; children would just mix with people from the same background:

“I think it’s good, it’s important because you’re, you and your family are one religion and one culture and if you’re only going to stick with that you’re not going to meet any other religion, but then if you come here and meet another religion, then they learn about it and that, so that’s another culture and they learn more about that instead of just knowing the one and sticking to the one, they know more about different ones and they can choose basically.”

(CFG 5S)

A similar sentiment was expressed by another child:

“I mean like some of the kids are like, they come from different countries and like this is their first-time spending time with like people from like Northern Ireland because usually they would just stay in their wee groups so it’s really good to see them come together,”

(CFG 4S)

One young person, speaking via a translator, described their experience of arriving in Northern Ireland the previous summer and how they had continued to attend the club which had helped them learn English and settle in the area:

“it was a very good experience to be a part of (holiday provision) programme from July until August. It was a good opportunity for us to learn English as that is the month we arrived or the month before we arrived in the country, so it gave us a bigger, a bigger idea of the area that we are living in, so we did, we got to learn some English”

(CFG 7S)

A peer mentor at another holiday club similarly reflected on how, when she had moved to Northern Ireland as a very young child, she had been unable to speak English, but being surrounded by the language in settings such as at school had helped her learn. Holiday clubs may potentially support children in language acquisition and development over the long summer holiday when schools are closed:

“it helps it helps them make new friends, helps them learn about new cultures and just helps them like gain their confidence in talking to people outside of their own community, it’s just helpful to them”

(CFG3S)

## 4. Discussion

In Northern Ireland, nearly one in five (*n* = 85,000) children live in relative poverty [15]. Although rates of child poverty have been relatively stable since falling from a peak of nearly one in four (*n* = 110,000) children in 2003, poverty amongst families with children is higher than in any other socio-demographic group in the country [15,16,17]. Moreover, in line with the rest of the UK, levels of child poverty in Northern Ireland are projected to increase in the next five years, largely as a result of reforms to the UK welfare and benefits system and the aftermath of the COVID-19 pandemic [18,19]. During the school summer holidays, families who are already living in poverty may face additional pressures: food shopping bills increase; the cost of keeping children entertained is prohibitive for parents and carers; and for working parents, the cost of childcare can be up to 25% higher compared to term time [5,6,7,20]. Furthermore, whereas the school summer holiday period in the state education sector in England, Scotland and Wales is typically six weeks long, the summer break in Northern Ireland is eight weeks long [21,22,23]. For the 102,000 children who normally receive free school meals during the school term, there is no state-funded support to enable children to access nutritious food during the summer holiday period, potentially placing them at risk of holiday hunger. An investigation of the holiday provision available in Northern Ireland found that dozens of organisations across the country have developed and delivered holiday provision enabling children to access food and activities during the summer break [4]. Clubs provided a range of enrichment activities, and some clubs were open five days a week so parents could work. However, in their analysis of holiday provision in Northern Ireland, Mann et al. (2018) [4] also found that holiday provision was fragmented and predominantly delivered by third-sector and community organisations that by and large, relied on a network of volunteers. Food provided in holiday club settings was varied and the majority of clubs could only provide snack-type foods. Moreover, two-thirds of the organisations delivering holiday provision ( *n* = 17) reported that they would struggle to provide more meals for attendees the following summer due to lack of resources. In response to the findings of the evaluation of holiday provision in Northern Ireland, Children in Northern Ireland part funded a small number of holiday clubs to pilot holiday provision in the country.

The aim of the current study therefore was to conduct a qualitative study regarding the delivery of a holiday programme from the viewpoint of those at whom the intervention is directed, namely children and young people. This was considered appropriate as it provides policy makers with a greater understanding and insight into how the implementation of a policy or intervention to support children is experienced by children and whether it has achieved its purpose and if not, why not [24]. Moreover, prior research suggests that interventions directed at children, and particularly vulnerable children, are more likely to make a difference to children’s lives if the voice of the child is incorporated into the co-development of the intervention [25].

Enabling children to access food in socially acceptable ways is a fundamental right that is enshrined in the United Nations Convention on the rights of the child [26]. Prior research has demonstrated that during the school summer holidays, children are at risk of experiencing holiday hunger, and that holiday clubs have been set up to help children access food and enrichment activities [2,3,6,27]. Children who attend holiday clubs are likely to come from food-insecure families and are highly likely to be living in areas of high socio-economic deprivation [6,28]. Older children and young people in the current study were very much aware that one of the objectives of holiday clubs was to provide support to children, particularly those who normally receive free school meals during the term, to access food during the holidays. They were also aware that parents try to shield their children from the financial difficulties that their families may be facing. Prior research has found that parents do their utmost to ensure that their children have enough food during the summer holidays and adopt a range of strategies to do this. Parents often sacrifice their own nutritional needs by reducing their own food intake, serving themselves smaller portion sizes, eating children’s leftovers or just not eating at all [5,8,29,30]. However, as demonstrated in the current study, children who live in food-insecure families are aware of the financial difficulties that their parents face, including the issue of not having enough food at home.

In the current study, primary school-aged children indicated that they were aware that some children had been referred to the holiday club, either through school or social services and as a result attendance fees had been waived due to family circumstances. They also spoke very candidly about the benefits of free food being provided in club settings and they felt that this equitable provision was important, suggesting that where costs were waived, it was handled sensitively and did not result in children feeling stigmatised. This attitude was very different to children’s self-reported experiences feeling shame and stigmatisation in receiving free school meals [31], and extends prior research with children on the benefits of holiday provision [8] as children were very much aware that holiday clubs served as a mechanism to alleviate food insecurity.

Echoing the findings of the only other peer-reviewed study regarding the benefits of holiday club provision from the perspective of child holiday club attendees [8], the current study also found that children perceived a number of additional benefits of holiday club attendance. Children and young people frequently spoke about how holiday clubs afforded opportunities to take part in a range of activities that they would not normally be able to access. Making and maintaining friendships was also a very important part of the holiday club experience for many children. Children and young people reported that holiday clubs provided them with a safe place, and this notion of safety enabled children to make new friends in a context that was inclusive and non-bullying. Prior research has similarly identified that children and parents are concerned about safety during the school holidays and are reluctant to let their children play outside [7], thus reinforcing the value of holiday provision as not only a social space, but a safe social space. When asked what they would be doing if they were not at holiday club, children and young people reported that they would probably be at home bored, watching TV or playing on computer games. The implications of a summer without holiday club may be potentially be detrimental to children’s health and well-being for a number of reasons. Studies in the USA have shown that during the summer holiday period children from low-income families are more likely to spend the summer at home alone, and watch TV for longer each day compared to children from higher-income households [32]. A recent study of the summer holiday experiences of more than 109,000 Welsh children and young people found that children from low-income households were more likely to go to bed hungry and were more likely to be lonely than children from higher-income households [33].

In addition to enabling children to access food, holiday provision provided opportunities for children and young people to gain important new life skills (e.g., CPR). Holiday provision also provided older children and young people with an opportunity to volunteer as peer mentors, supporting their progression towards achieving a qualification in Youth Leadership. Children and young people also reported that their experiences at holiday club boosted their self-confidence. Research suggests that participation in extra-curricular activities such as volunteering not only boosts confidence, but it also improves self-esteem, self-awareness, social skills and ultimately academic achievement [34]. Cumulatively, these additional benefits of holiday club provision identified by young people and children have the potential to have a positive impact on their mental health and well-being and life chances.

Children and young people in the current study also considered that holiday clubs benefited parents in numerous ways. They were aware that the school holidays are an expensive time of year for parents, echoing the findings of prior research with adults that demonstrates that childcare costs increase during the school holidays and low-income parents and carers often struggle to meet the extra demands placed on their already limited resources [6,29,30,35]. In the current study, some clubs charged for attending but waived attendance fees for children who had been referred to the scheme by social services of schools. However, it appears that this process was handled sensitively and did not result in children feeling stigmatised or embarrassed. Even where the cost of attending was not waived, both younger and older children considered that the cost of attendance represented good value for money as the relative cost of childcare was much higher. There are numerous studies that show that the availability and cost of childcare during the school holidays in the UK are problematic. Firstly, there are not enough places available and where places are available, the cost of childcare provision across the holiday period is significantly higher, costing as much as 25% more compared to term time [20]. Secondly, for families in the UK who rely on Universal Credit, the requirement to pay childcare fees in advance and then reclaim them through the benefits system means that some low-income parents cannot afford to work during the summer holidays [36,37].

The cost of childcare, however, is just one of the many competing demands placed on low-income parents’ finances during the summer. There are increased costs in keeping children entertained, and food shopping bills increase due to children being at home, often resulting in household bills not being paid on time or not paid at all [6,30,35]. Children and young people in the current study considered that attendance at holiday club reduced parental anxieties about household finances and feeding their children during the summer holidays, reflecting the findings of a pilot study by Long et al. (2018) [6]. In addition, children and young people suggested that holiday clubs also provided parents and carers with some respite and some time for themselves. Children’s perceptive views on the potential benefits of holiday provision for parents and carers reflected the quantitative findings of studies that have shown that have shown that holiday programmes have the potential to reduce household food insecurity and to support parents’ and carers’ mental and emotional well-being [7].

Children from both age groups spoke positively about the way their clubs operated and found it difficult to make recommendations on how clubs could be improved. Children indicated that they would like an increase in the number and range of activities provided, and increased opportunities for free play, breaks, and day trips. The discussion regarding the provision of food produced mixed responses that appeared to be based on children’s food preferences or familiarity with certain types of foods. For example, children readily ate ‘toasties’ and snack foods but did not consume homemade soup. This is an important issue as providing children with healthy, nutritious food is one of the key objectives of many holiday programmes [4]. Numerous studies demonstrate that the quality of an individual’s diet during childhood can have serious implications for health in adulthood, with a poor diet associated with poorer health and behavioural outcomes [38,39,40]. Multiple factors influence children’s eating behaviours including parental modeling of eating behaviours and feeding styles, structure, routine and sources of food [41,42]. It may be the case that holiday clubs present an opportunity for children to experience and become familiar with new food items and improve their eating behaviours [43,44]. Difficulties in sourcing food and a lack of facilities to prepare food, accompanied by a heavy reliance on volunteers, were identified as key barriers to extending provision of holiday clubs in Northern Ireland [4].

Finally, older children reported how holiday clubs benefitted the wider communities that they serve. Secondary school-aged children were aware that holiday club provision had the potential to bring communities together. Holiday clubs could, they suggested, play an important role in bringing together members from different religious and cultural backgrounds. We argue that the findings in this paper show that holiday clubs provide an important resource for children, families, and the communities that they serve.

## 5. Conclusions

To conclude, therefore, the findings of this study highlight that children and young people are acutely aware of the structural causes of poverty and associated outcomes, such as food insecurity, and a lack of disposal household income on multiple actors. Children and young people view holiday programmes as a means of alleviating the negative impacts of poverty for parents and children but are also aware of the additional benefits holiday clubs provide to communities. Moreover, the findings of the current study provide a unique insight into how holiday provision provides a means of bringing together children from across different cultural and religious backgrounds. This is particularly important given Northern Ireland’s history of sectarian divisions but is also applicable in the context of newly arrived immigrant populations in the country. Whilst this study has provided a useful insight into the views of children regarding holiday clubs in Northern Ireland, it is important to acknowledge these data were only collected from a small number of clubs and therefore may not be generalisable to wider geographic areas of the UK. Furthermore, this study does not represent the view of parents and staff, which may differ to those of the children.

The findings of this study also have implications for local and national policy makers in terms of involving children and young people in decision making. A number of studies suggest that public participation and influence provides additional knowledge within local decision making [45,46], often leading to co-designed solutions [47]. Involving the public and user groups in policy decisions and implementation of policies not only improves collective awareness of local needs [48] but also results in positive outcomes for communities (e.g., Brunton et al. 2017) [49] Likewise, many studies have reported positive benefits to individuals involved in participatory initiatives in terms of well-being [50]. However, the evidence of involving disadvantaged communities in decision-making processes and reducing social, health and educational inequalities is rather mixed [51,52]. Although we acknowledge that further research is required to explore the role of children and young people in local and national decision making, we argue that the findings of the current study suggest that local authorities and government should seriously consider listening to the voices of children and young people in policy decision-making processes.

## Figures and Tables

**Table 1:** Demographic information for child participants.

Participants Total Sample	Club 1		Club 2		Club 3		Total	
Number of children [*n* (%)]	14 (23%)		47 (71%)		4 (6%)		65	
Age	4–11	12–17	4–11	12–17	4–11	12–17	4–11	12–17
**N (%)**	8 (57)	6 (43)	22 (47)	25 (53)	4 (100)	0	34 (52)	31 (47)
Gender								
Male (N (%))	3 (38)	2(33)	4 (18)	13 (52)	2 (50)	0	9	15
Female (N (%))	5 (62)	4 (67)	18 (82)	12 (48)	2 (50)	0	25	16

**Table 2:** Characteristics of holiday clubs and site rankings in the Northern Ireland Index of Multiple Deprivation.

Club	Hours	Age Range	Days	Charge	No. of Weeks	Meals and Type of Food	Activities	* Northern Ireland IMD 2017
1	9.30 a.m.–2.30 p.m.	4–17	Monday–Wednesday	Free	3	Breakfast and lunch: cereal, toast and cooked lunch and dessert	Physical activities, arts and crafts, kitchen activities, mini medics, OCN in Youth Leadership	412
2a	10.00 a.m.–12.00 noon	4–7	Tuesday, Wednesday, Friday	£2.00 per day but waived if child or young person referred by school or social services	3	Breakfast, lunch and supper: snack-type food, supper	Physical activities, arts and crafts, mini medics, outings, OCN in Youth Leadership	115
	1.00 p.m.–4.00 p.m.	8+	Monday–Friday		3			
	7.00 p.m.–10.00 p.m.	Teen club	Monday–Friday		4			
2b	12 noon–3.00 p.m.	7+	Monday–Friday		3			
3	9.30 a.m.–3.00 p.m.	4–12	Monday–Friday	£1–£5 depending on activity	5	Fruit, sandwich lunch	Physical activities, outings, dancing, mini medics and outdoor activities	127

* Information from the Northern Ireland Index of Multiple Deprivation (Rank: 1–890). All 890 small areas in Northern Ireland have been ranked according to a range of indicators including income, employment, health, crime, housing, education and access, with the most deprived having a rank of 1 and the least deprived a rank of 890.

**Table 3:** Schedule of prompts for children’s focus groups.

Children’s Focus Group Schedule of Prompts.
Why did you decide to come to the holiday club? What do you do at the holiday club? Could you tell me more about what activities you do at the club? Who decides what activities you do at the club; do you get to pick? What do you think about the activities at the holiday club? Do you enjoy the activities you do at the club? Have you learnt anything from attending the clubs? What is your favourite activity that you do at the club? What food and drinks do you have at the holiday club? Who decides what food and drink you have at the club, can you pick? Do you enjoy the food and drinks served at the holiday club? What is your favourite food and drink at the holiday club? Do you enjoy getting to eat and drink at the holiday club, if so, what do you enjoy about it? Overall, what do you like best about coming to the holiday club? If you had to tell another child about the holiday club who had never been before, what would you tell them? How does the holiday club make you feel? How would you feel if you did not have the holiday club to attend during the summer? Have you made any new friends from attending the holiday club? Do you see any friends you already had more during the holidays from attending the holiday club? Is the holiday club easy for you to get to? Do you enjoy having the holiday club here or do you think it should be at a different place? If so, why do you like having club here? If not, where and why? Do your parents have the opportunity to attend the holiday club? If yes, why do they come along and what do they do at the club? If no, would you like it if they were able to come along? What do your family think about the holiday club? Do you think the holiday club makes any difference to your family in the holidays? If so, why and how? What activities would you do if you did not come to the holiday club? Are these activities different to what you do at the holiday club, if so, how? What kind of food would you eat if you did not attend the holiday club? Is this food any different to what you have at the holiday club, if so how? Do you eat any new or different food at the clubs? What is your favourite meal at home and what kinds of meals do you eat most at home? Do you eat three meals a day at home [breakfast, dinner and tea]? What kinds of meals do you eat most at the holiday club? What do you not enjoy about the holiday club? If anything, what do you think would prevent you from coming along to the club? What improvements could be made to the holiday club to make the club even better? Would you make any changes to the meals or activities at the holiday club? If so, what and why? Is there anything else you would like to mention which we have not covered?

**Table 4:** Sub-themes identified in interviews with children and young people according to age group.

Theme	Sub-Themes	Age Group
The need for holiday clubs	Holiday hunger; family circumstances.	Primary school-aged children Secondary school-aged children
Benefits of holiday provision for children and young people	Activities; skills and confidence; fun and friendship; safe place, alternative to holiday club attendance	Primary school-aged children Secondary school-aged children
Benefits of holiday provision for parents	Expense of summer holidays; saving parents money; respite; reduce parental anxiety about food	Primary school-aged children Secondary school-aged children
Operational characteristics of holiday clubs	Type of activities, length of provision and hours of operation and food	Primary school-aged children Secondary school-aged children
Benefits of holiday provision for individuals, families and communities	Community cohesion; removing barriers; supporting child immigrants	Secondary school-aged children

## Data Availability

The data presented in this study are available on request from the corresponding author. The data are not publicly available to prevent identification of participants.
